# SAfE transport: wearing face masks significantly reduces the spread of COVID-19 on trains

**DOI:** 10.1186/s12879-022-07664-0

**Published:** 2022-08-17

**Authors:** Hanna Grzybowska, R. I. Hickson, Bishal Bhandari, Chen Cai, Michael Towke, Benjamin Itzstein, Raja Jurdak, Jessica Liebig, Kamran Najeebullah, Adrian Plani, Ahmad El Shoghri, Dean Paini

**Affiliations:** 1 Data61, CSIRO, Sydney, Australia; 2 Health and Biosecurity, CSIRO, Sydney , Australia; 3 College of Public Health, Medical and Veterinary Sciences, and Australian Institute of Tropical Health and Medicine, James Cook University, Townsville, Australia; 4 Transport for New South Wales, Sydney, Australia; 5 Queensland University of Technology, Brisbane, QLD Australia; 6 Research Centre for Integrated Transport Innovation,School of Civil and Environmental Engineering, University of New South Wales, Sydney, NSW Australia

**Keywords:** COVID 19, SARS-CoV-2, Transit assignment, Disease spread model, Face masks

## Abstract

COVID-19 has had a substantial impact globally. It spreads readily, particularly in enclosed and crowded spaces, such as public transport carriages, yet there are limited studies on how this risk can be reduced. We developed a tool for exploring the potential impacts of mitigation strategies on public transport networks, called the Systems Analytics for Epidemiology in Transport (SAfE Transport). SAfE Transport combines an agent-based transit assignment model, a community-wide transmission model, and a transit disease spread model to support strategic and operational decision-making. For this simulated COVID-19 case study, the transit disease spread model incorporates both direct (person-to-person) and fomite (person-to-surface-to-person) transmission modes. We determine the probable impact of wearing face masks on trains over a seven day simulation horizon, showing substantial and statistically significant reductions in new cases when passenger mask wearing proportions are greater than 80%. The higher the level of mask coverage, the greater the reduction in the number of new infections. Also, the higher levels of mask coverage result in an earlier reduction in disease spread risk. These results can be used by decision makers to guide policy on face mask use for public transport networks.

**Supplementary Information:**

The online version contains supplementary material available at 10.1186/s12879-022-07664-0.

## Introduction

In December 2019, a local Wuhan (Hubei, China) outbreak of severe acute respiratory syndrome (SARS-CoV-2), causing the disease referred to as COVID-19, became a pandemic. It has spread to 222 countries, with over 191 million confirmed cases and more than 4.1 million deaths to date [43]. The result has been a massive impact on the global economy, including government and individual debt; a slowdown in national and international travel, tourism, commerce, and trade; and sociological issues (see, for example, [9, 37]). It has effectively challenged the status quo of day-to-day activities and ways of life. Another, more specific, example is the change in the way people commute and use services such as public transport. Initially, the substantial drop in public transport patronage was due to local or national lock-downs. However, with restrictions lifted in some countries, there has generally not been a concurrent increase in public transport use [16]. Globally, people are reluctant to use public transport for fear of contracting COVID-19. With the emergence of potentially more virulent COVID-19 variants that could reduce the effectiveness of vaccinations, it is of paramount importance that public transport operators are able to ensure their services are safe in order to restore public confidence.

There are various non-pharmaceutical measures that can be applied to increase safety on public transport. For example, these include but are not limited to: the introduction of more thorough and more frequent cleaning of the rolling stock; protecting the drivers and public transport operators with personal protective equipment (PPE); mandating wearing face masks by passengers; marking seats and standing spaces to maintain physical distances; forbidding front-door boarding to protect drivers and encourage boarding through other doors (particularly on buses [21]); lowering the maximum occupancy limits; encouraging hand sanitation; and shutting down stations shown to be hot-spots.

For researchers, the challenge is to show that these measures can be effective at reducing the spread of COVID-19 on public transport networks. Indeed, there has been research that shows the survival time of the virus on different surfaces [31], the effectiveness of cleaning [8, 13], hand sanitation [15, 32], and lowering maximum occupancy limits [11, 21, 33]. Perhaps the most obvious measure would be the mandating of face masks for commuters, and while face masks have been generally accepted by most to be able to significantly reduce the spread of COVID-19 (see, for example, [7, 12, 18, 22, 38, 40]), no work has attempted to quantitatively estimate the benefit of face masks on public transport. The benefit of such work could provide substantial reassurance to both government policymakers charged with ensuring the public’s safety when they commute and public commuters themselves.

Here we present a tool that can explore the probable impacts of such measures to better support strategic and operational decision-making by public transport operators. The Systems Analytics for Epidemiology in Transport (SAfE Transport) tool combines an agent-based transit assignment model, a community-wide transmission model, and a transit disease spread model. The tool has been tested on a set of artificial scenarios exploring the potential impact of non-pharmaceutical mitigation strategies such as face masks, using COVID-19 realistic values reported on other train networks. We focus on a case study of COVID-19 on the train network in Sydney, Australia, exploring the probable impacts of different proportions of passengers wearing face masks (that is, population level face mask coverage).

## Results

To determine the probable impact of face mask wearing coverage by passengers, we focus on the total number of new COVID-19 infections over a 7-day time period as the output of interest. That is, the cumulative number of new infections over a 7-day simulation time horizon. We consider the following scenarios:

- Baseline: no mitigation strategies were applied (baseline)
- Face masks are worn by 25% of commuters (Mask_25).
- Face masks are worn by 50% of commuters (Mask_50).
- Face masks are worn by 75% of commuters (Mask_75).
- Face masks are worn by 80% of commuters (Mask_80).
- Face masks are worn by 100% of commuters (Mask_100).

To account for stochasticity in the underlying disease transmission process, each scenario was run 100 times, with summary statistics provided for the average and variation across all of these repeats. For each scenario, the relevant proportion of passengers were selected at random to wear a mask, independent of their disease progression status (that is, susceptible, exposed, infectious, or recovered). Further information is provided in "Methods" and the Additional file 1.

### 80% of passengers need to wear a mask to see a statistically significant reduction in 7 days

**Fig. 1:**
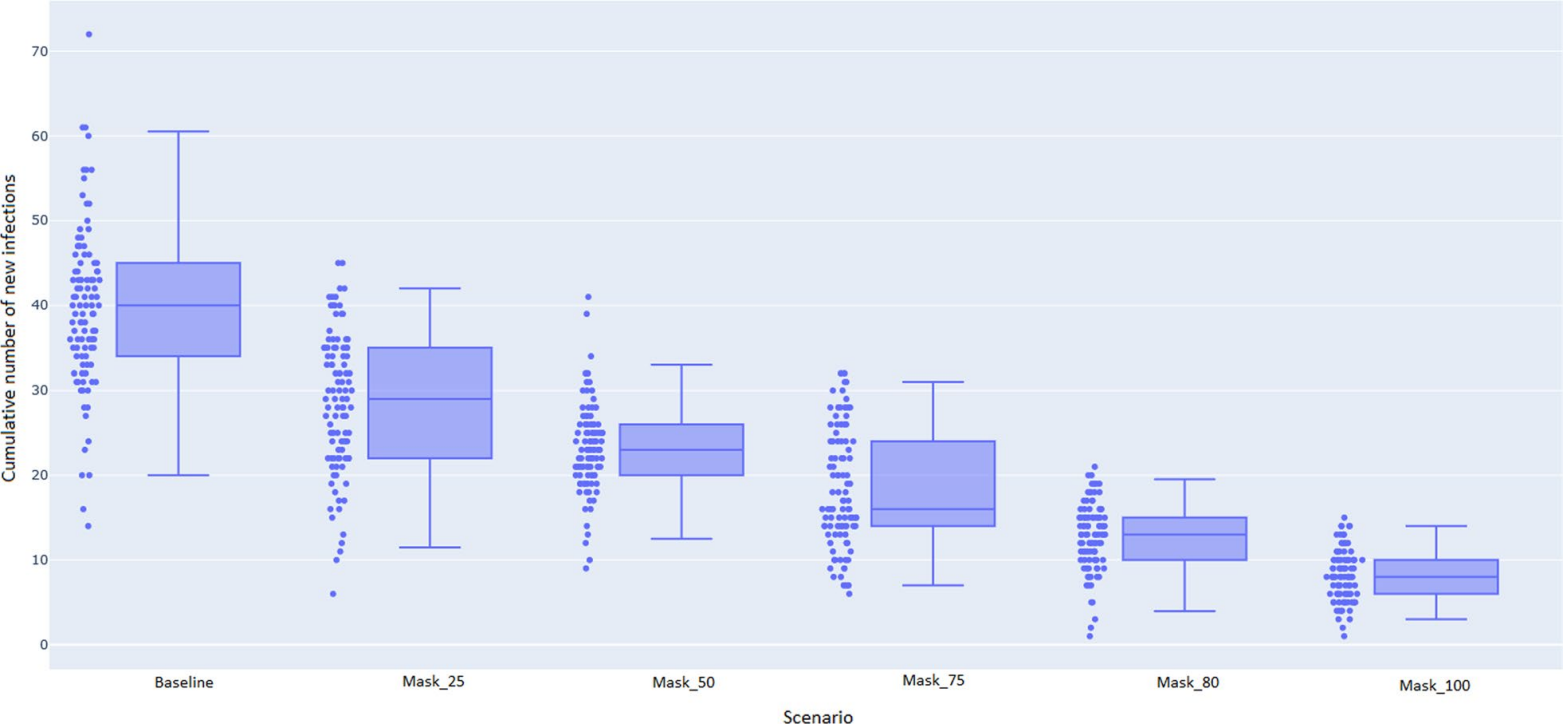
Total number of new infections after 7 days for the different mask wearing coverage scenarios Note: here the whiskers depict the 95% confidence intervals

The probable impacts of the different face mask wearing coverage levels over the full 7-day horizon are summarised by the Box Plot depicted in Fig. 1. Note that, for ease of visually identifying statistically significant differences, the whiskers display the 95% confidence intervals, as opposed to the traditional minimum and maximum. That is, if the top whisker of the mask wearing scenario is below the bottom whisker of the baseline, that level of mask wearing coverage is statistically significant over the course of the considered time horizon of 7 days. We demonstrate that the scenario with 80% of passengers wearing face masks shows a 68% reduction in the total number of new infections, and this is statistically significant when compared to the baseline of no masks. In the case when 100% of passengers wear face masks, the reduction is 80% in comparison with the baseline with no masks.

An important aspect of this model is that face masks do not perfectly prevent viral shedding (transmission from an infectious person) or infection of those susceptible. This is reflected by the fact that there are still new cases despite 100% mask wearing coverage.

### Divergence between the baseline and mask wearing scenarios increases over time

**Fig. 2:**
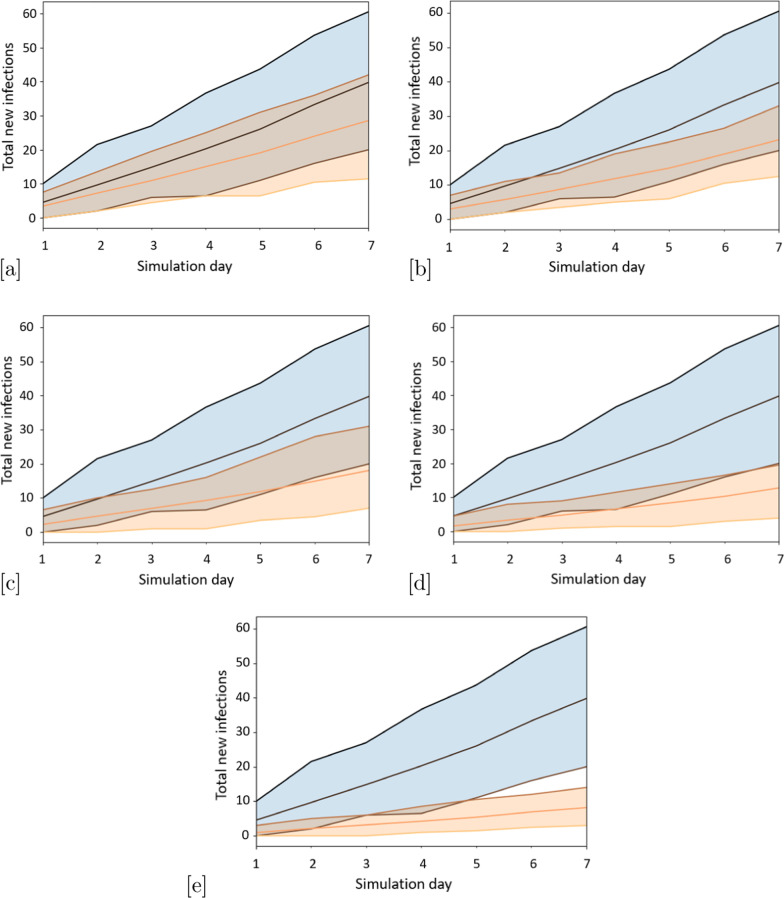
Comparison of the evolution in time of the total number of infections between the baseline (blue) and scenarios with face masks (orange) (average and 95% confidence intervals): **a** baseline vs Mask_25, **b** baseline vs Mask_50, **c** baseline vs Mask_75, **d** baseline vs Mask_80, **e** baseline vs Mask_100.

Figure 2 shows the total number of infections over time for all the mask wearing scenarios in comparison with the baseline. As the percentage of people wearing masks grows, the impact becomes more obvious over the 7-day time horizon. The higher the percentage of people wearing face masks, the sooner the results diverge significantly. For the 80% scenario, statistical significance does not occur until day 7, while for the 100% scenario, this occurs on day 5. For a 7-day time frame, then, the 80% scenario may be defined as a “tipping point”. For longer simulation horizons, the two compared distributions are expected to continue diverging, and after enough time, even the 25% scenario may become statistically significantly different from the baseline.

We have focused on a 7-day time horizon as a period when the general public would like to see positive improvement from expectations that they wear masks. However, this indicates that for more strategic planning, longer time horizons may be of interest.

### How robust are the mask wearing effects on transmission proportions via direct vs. fomite routes?

**Fig. 3:**
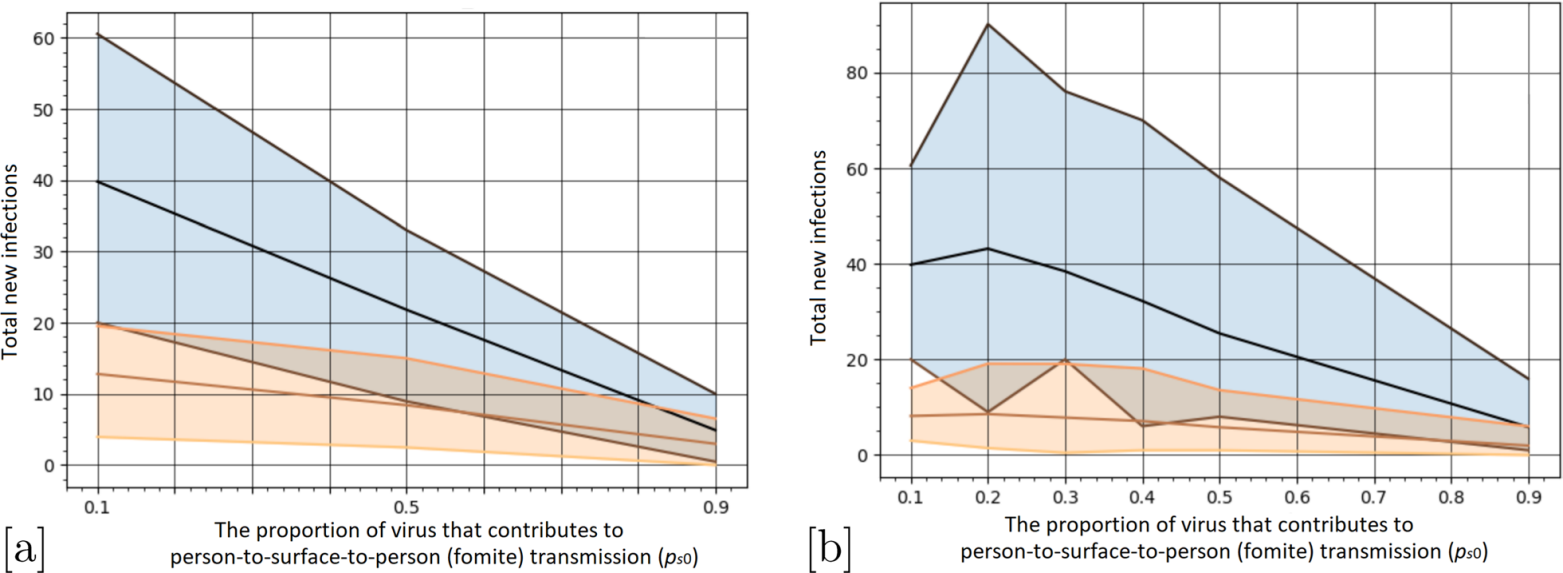
Comparison of how the total number of infections is affected by the proportion of virus contributing to fomite transmission in the absence of a mask ($p_{s0}$) (in blue). The lines depict the average, and the shaded region indicates the 95% confidence intervals based on 100+ simulations; **a** 80% mask coverage, **b** 100% mask coverage (in orange). Note: for $p_{s0}=0.2$ and 0.3, there are 200 repeat simulations to capture the stochastic variation

One of the key transmission model parameters is the proportion of virus that contributes to person-to-surface-to-person (fomite) transmission ($p_{s0}$), versus person-to-person (direct) transmission ($1-p_{s0}$). Figure 3 depicts how the average number of total infections after 7 days is affected by this parameter. The current evidence suggests aerosol transmission likely dominates (see, for example, [14, 17, 26, 35]), and so the proportion of virus shed by infectious passengers that contributes to fomite transmission when no mask is worn is expected to result in small values of $p_{s0}$. We find that 100% mask coverage results in a statistically significant reduction of new cases after 7 days when $p_{s0}\lessapprox 0.125$ (Fig. 3b). Further, we find 80% mask coverage only results in a statistically significant reduction in the average number of new cases after 7 days for values of $p_{s0}\le 0.1$ (Fig. 3a).

## Methods

**Fig. 4:**
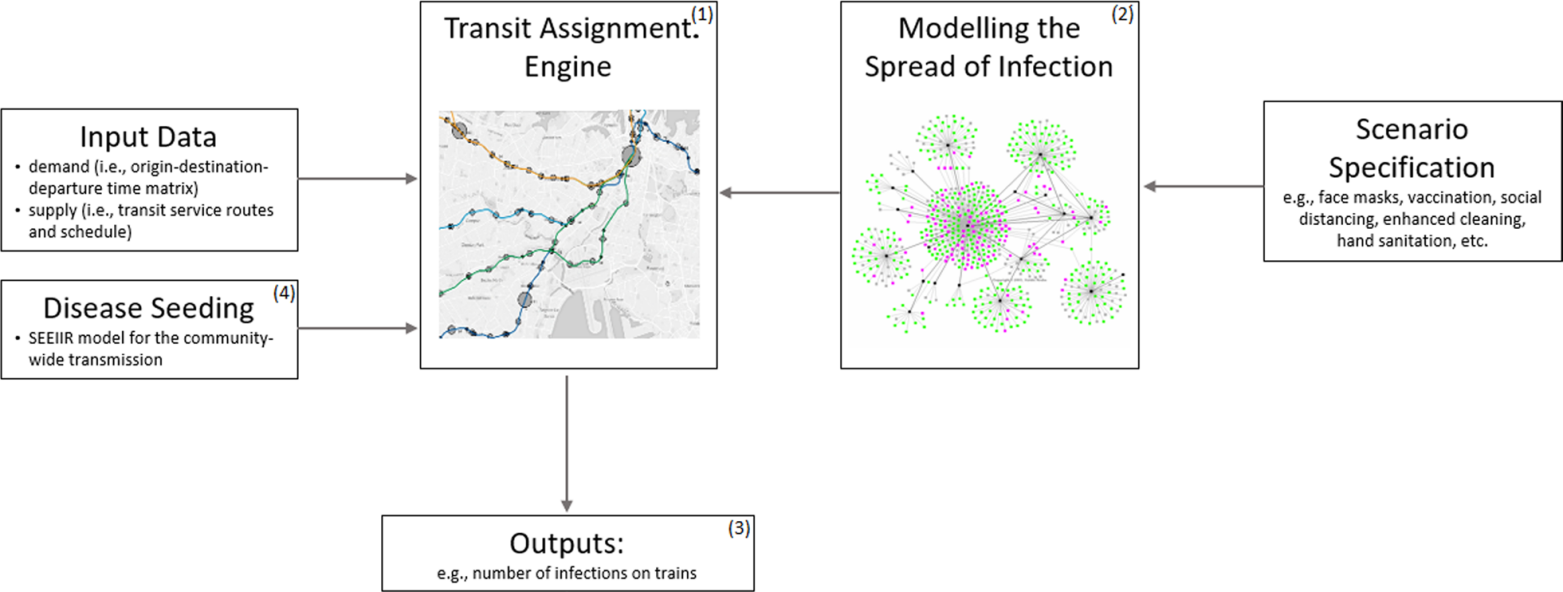
The modelling framework, with more information on each component outlined in "Transist assignment engine"–"Seeding". The modular structure allows for flexibility in the designation of geographical location, pathogen of interest, and scenarios explored. The agent-based model yields detailed outputs to inform operational and strategic decisions

We developed a modelling framework to capture the key components of pathogen transmission on public transport networks. This is a general framework that could be used for a wide variety of pathogens, transport modes (including multi-modal) and public transport networks, though here we focus on COVID-19 transmission on trains. An overview of the modelling framework is provided in Fig. 4. There are four main components:

1. Transit assignment engine: a transit assignment engine simulating the movement of passengers in the public transport network. For our case study, this is the train network in Sydney, Australia.
2. Modelling the spread of infection: the transit disease spread model. For our COVID-19 case study, this incorporates two forms of transmission: direct (person-to-person) and fomite (person-to-surface-to-person).
3. Outputs: an analytical module providing summary statistics and visualisations of results.
4. Disease seeding: a community-wide transmission model that informs disease seeding (the expected number of infectious passengers on the Sydney train network).

In addition to these components, our modelling framework requires input data and specification of the scenarios of interest (such as including any non-pharmaceutical interventions being considered).

In our case study, this framework involves assigning a passenger to a train trip leg based on travel smart card data and an assigned shortest feasible path. Each leg of the trip is assigned to a transit service vehicle (in this case, a train), according to a specified capacity and current occupancy. The trains operate in accordance with a pre-defined schedule. Every time a passenger finishes their trip, the transit disease model is triggered and this passenger is assigned an exposure status (that is, whether the passenger was infected during their trip). The information about new infections is sent to the visualisation module to be displayed on a dashboard. At the end of every simulated day and at the end of the simulation horizon, the total number of new infections is calculated and reported for final evaluation. The SAfE Transport modules are further described in "Transist assignment engine"–"Seeding".

### Transit assignment engine

The transit assignment engine is an agent-based simulation platform that maps trip demand to transit service supply. The transit assignment engine underpins SAfE Transport, as it provides the network of contacts between travelling passengers (that is, with whom and for how long they are in contact).

The engine depends on input data in the form of:

1. trip demand (that is, trip origin, trip destination, and starting trip time for every passenger), and
2. transit service supply (that is, routes and schedules of public transport services).

The full architecture is provided in the Additional file 1. The total number of trips on Sydney’s train network before COVID-19 was about 8 million per week. To account for the patronage drop during COVID-19, we assume the demand to be 10% of the total, which aligns with the real-life observations [27, 29, 30]. The supply of services did not change in Sydney due to COVID-19. Trip demand is captured by smart cards, including tap-on and tap-off data with location GPS coordinates and timestamp. This is used by the shortest path router to calculate a set of time-dependent shortest paths for each travelling passenger using the classic Dijkstra algorithm. Throughout the simulation, passengers are tracked and detailed dynamic outputs are collected for every agent, link (between two consecutive stops/stations), stop/station, service vehicle, service line, and the whole network.

### Modelling the spread of infection on trains

At its core, the disease spread model on trains provides a probability of becoming infected for each susceptible passenger, based on the current and past travel of infectious passengers in the same spatial area. The model uses a number of simplifying assumptions, the most important being that we ignore any age-based effects (all agents are identical). We also assume homogeneity of mixing within the spatial area considered, including an equal distribution of passengers throughout the train. To account for our homogeneous mixing within a spatial area assumption, we use a half-carriage spatial area for our model, due to the stack structure (including upper and lower deck) of the Sydney train carriages (Waratah design).

The overall probability of a susceptible individual being infected is based on the standard probabilistic statement of being one minus the probability that they were not infected. This allows us to consider the probability of infection from each of the infectious passengers, and the surfaces on the half-carriage, separately. The transmission model uses the regression expression for the attack rate from the study by Hu et al. [19] (Fig 4 in [19], Average of all seats), both directly for the empirical person-to-person part of our transmission model, and for calibration of the mechanistic person-to-surface-to-person model. The mechanistic model is based on the transmission route model developed by Atkinson and Wein [1]. This approach models proportion of the virus shed drops to the surface, and the effective dose based on surface concentration. We then use a standard dose-response model for the probability of infection from contaminated surfaces (fomites). Further details of the transmission model are provided in the Additional file 1, along with sensitivity analyses of key parameters.

### Seeding

Our primary disease spread model, as outlined in "Modelling the spread of infection on trains" only considers transmission on the public transport network (and is specifically calibrated to trains). To keep the numbers of infectious passengers travelling on trains identical across simulations for comparability, we use a deterministic compartmental model to approximate the transmission dynamics in the general community.

We use the standard susceptible-exposed-infectious-recovered progression structure, with the exposed and infectious compartments repeated to better account for the distribution of time spent in those states (see, for example, [20]). We refer to this as an “SEEIIR” model for short. We start the deterministic SEEIIR model with 2000 infectious cases, using the population of Sydney of 5.73 million in 2019 [4], and a basic reproduction number of 2.5 [6, 23, 45]. We used estimates of the proportion of the population that commutes via public transport (approximately 20% according to Census 2006, 2001 and 2016 data [2, 28]), and the proportion of commuters who use trains (approximately 50.9% [39]), to arrive at an estimate of 10% of the population using trains in Sydney. Further details are provided in the Additional file 1, including a table with the numbers used to seed infectious passengers for each of the 7 days.

### Face mask wearing scenarios

The primary objective of this work was to explore the probable impacts of different face mask wearing proportions by passengers (that is, face mask coverage). How the mask wearing status of passengers effects the transit transmission model depends on whether the passenger is susceptible or infectious. The mask wearing status of infectious individuals reduces viral shedding. The mask wearing status of susceptible individuals is known to reduce the overall probability of infection [38], but does this through two separate mechanisms: (i) it explicitly effects the probability of being infected from the direct transmission component; and (ii) it effects the effective dose from the surface for the fomite transmission component.

To parameterise the viral shedding effect on those infectious passengers, we use the filtration efficacy of a two-layer cloth mask, as described in Howard et al. [18]. They discovered that two-layer cloth masks reduce infectious particle load by 88-94% and have a filtration efficacy of 80-90%. In our modelling, we use 90% as it is within both of these bounds, and reduction in infection particle load is arguably the most important aspect.

For the reduction in susceptibility, the model was calibrated using a minimisation of the sum of squared errors to achieve the reported odds ratio of 0.22 for wearing a mask [38]. There is further information on this provided in the Additional file 1.

## Discussion

The SAfE Transport tool presented here can support public transport agencies in both strategic and operational decision-making for disease spread mitigation options. Here we have applied it to train networks for informing COVID-19 related mitigation options. Hence, the SAfE Transport tool is capable of contributing to recovery and resilience post-pandemic.

We have shown that for this parameterisation of COVID-19 transmission on trains, if more than 80% of passengers wear face masks, there is a substantial (68% relative) and statistically significant reduction in the total number of new cases over a 7-day period. While this result is highly sensitive to the proportion of virus shed contributing to fomite vs direct transmission, recent evidence [17, 35] suggests fomite transmission is only a small contributor, and that indeed the proportion is speculatively around 10% (equivalent to the value of $p_{s0}=0.1$, used here), and likely to be even smaller [14]. This finding and subsequent sensitivity analysis demonstrates the utility of our SAfE Transport tool for a nuanced way of informing decision-making with respect to disease spread on public transport networks.

While we made a number of simplifying assumptions with respect to the disease transmission models, we explored the effects of most of these assumptions. The effect of the key parameter of the model on our outcome of interest, the total number of new cases, is shown in "How robust are the mask wearing effects on transmission proportions via direct vs. fomite routes?", and the rest are shown in the Additional file 1. We assumed masks worn had the efficacy of a two-layer cloth mask. However, in the case when mask wearing has been mandated, even if some people are wearing less effective masks, we would still expect to see a substantial and statistically significant reduction in new cases since our threshold for significance is 80%. Our transmission model does not explicitly explore aerosol transmission, but this is implicitly included in the direct and fomite transmission being calibrated to recover the attack rate on trains reported by Hu et al. [19]. One major assumption made and not tested was the lack of age structure, which we ignored since our focus here has been on the use of adult commuters as primary train users [36].

Although our study was limited to trains (specifically, a half-carriage), policymakers and public transport operators could confidently mandate the use of face masks on other, similar, modes of public transport to help reduce COVID-19 spread. A similar effect is expected for other public transport modes such as buses and trams, given their similar physical attributes. For example, the sitting areas in public transport modes are organised in a similar pattern and proximity within a closed space.

Maintaining the recommended distance of 1.5 metres between passengers [5] on any public transport mode would be difficult if travel demand returned to pre-COVID-19 levels. However, we would expect that while face masks would be mandatory in that situation, it would not be the only public health measure used to reduce the spread of COVID-19, as even with 100% of passengers wearing masks, we still show a low spread of COVID-19. We therefore strongly support the “Swiss cheese” approach to mitigation and control efforts (see, for example, [25]).

In its design, the proposed modelling framework for SAfE Transport is generic and agnostic to transport networks and travel modes. It effectively enables the development of a digital twin of any public transport network (and any transport mode), providing a holistic view of its performance (for example, identifying hot-spots) and the dynamics of a contagious disease spread.

## Conclusion

In conclusion, we have developed a flexible tool for supporting operational and strategic decision-making for disease transmission mitigation measures on public transit networks. The SAfE Transport tool is fast-performing and highly efficient, while its open architecture allows for the integration of complementary data sets to enhance the level of detail and modelling accuracy. The agent-based modelling allows a fine-grained representation of agents’ behaviour and interactions, and close dynamic tracking facilitates time-dependent studies. The SAfE Transport tool allows for building a digital twin of any transport network in any city, representing various modes of transport (including multi-modal), modelling various transmissible diseases and allowing for exploratory scenarios. This provides an opportunity for a multitude of further investigative studies.

The scientific fundamentals and applicability of SAfE Transport have been validated in this case study, which demonstrates that mask wearing substantially reduces impact on COVID-19 transmission, with at least 80% coverage levels in a 7-day time period. We also found that higher levels of mask coverage result in an earlier and larger reduction in disease spread risk.

There are still many unanswered questions and avenues of research with regards to reducing the spread of COVID-19 on public transport networks and restoring public confidence. Our SAfE Transport tool has significant potential, both in helping to answer specific COVID-19 questions and also for other infectious diseases. In particular, the SAfE Transport tool is designed for a nuanced approach to informing operational and strategic decision-making for public transport networks. Other areas of consideration that could be investigated with the SAfE Transport tool include: (i) understanding how cleaning regimes affect the transmission dynamics (and how sensitive this is to the proportion of transmission that is direct vs. fomite), (ii) understanding how other COVID-19 variants, such as Delta, might be affected by non-pharmaceutical strategies; (iii) determining general transmission patterns and, in particular, hot-spots on the network and how they are affected by different measures; (iv) explicit modelling of other public transport types to confirm the applicability of our findings across general networks; (v) better understanding spatial distribution of passengers within public transport vehicles (such as carriages for trains) and effects of different passenger densities; (vi) exploring longer simulation time horizons; and perhaps most importantly, (vii) including community vaccination levels to determine the need or effectiveness of non-pharmaceutical strategies.

## Supplementary Information

**Additional file 1: **The Supplement to SAfE Transport: Wearing Face Masks Significantly Reduces the Spread of COVID-19 on Trains provides additional detailed information on the transit assignment engine's full architecture, the transmission model on trains, seeding, modelling assumptions and parameters, and sensitivity analysis, including dose response calibration and the number of repeats required to capture stochastic variance.

## Data Availability

For data and material-related inquiries, please contact the corresponding author: Hanna Grzybowska.
