# Supplementary material for: SAfE transport: wearing face masks significantly reduces the spread of COVID-19 on trains
Source: BMC Infect Dis. 2022 Aug 17;22:694. doi: 10.1186/s12879-022-07664-0 (PMC9382008; doi:10.1186/s12879-022-07664-0)
Supplement: Supplementary file 1 — Additional file 1: The Supplement to SAfE Transport: Wearing Face Masks Significantly Reduces the Spread of COVID-19 on Trains provides additional detailed information on the transit assignment engine's full architecture, the transmission model on trains, seeding, modelling assumptions and parameters, and sensitivity analysis, including dose response calibration and the number of repeats required to capture stochastic variance. [file 12879_2022_7664_MOESM1_ESM.pdf]

Supplement to SAfE Transport: Wearing Face Masks Significantly Reduces the Spread of COVID-19 on Trains

Hanna Grzybowska[a,f,*], R.I. Hickson[b,c], Bishal Bhandari[a],
Chen Cai[a], Michael Towke[d], Benjamin Itzstein[a], Raja Jurdak[e], Jessica Liebig[b],
Kamran Najeebullah[a], Adrian Plani[a], Ahmad El Shoghri[b], and Dean Paini[b]

[a] Data61, CSIRO, Australia
[b] Health and Biosecurity, CSIRO, Australia
[c] College of Public Health, Medical & Veterinary Sciences,
and Australian Institute of Tropical Health and Medicine,
James Cook University, Townsville, Australia
[d] Transport for New South Wales, Sydney, Australia
[e] Queensland University of Technology, Brisbane, QLD, Australia
[f] Research Centre for Integrated Transport Innovation,
School of Civil and Environmental Engineering,
University of New South Wales, Sydney NSW, Australia
[*] Corresponding author

1 SAfE Transport

1.1 *Transit assignment engine*

The transit assignment engine is an agent-based simulation platform that allows us to understand the network of contacts between travelling passengers (with whom and how long they stay in contact with) – a key input to modelling the spread of infection in public transport networks. Its architecture is presented in Figure 1.

The transit assignment engine requires input data on: (1) trip demand (that is, trip origin, trip destination, and start trip time for every passenger); and (2) transit service supply (that is, routes and schedules of public transport services). The total number of trips on Sydney's train network before COVID-19 was about 8 million per week. To account for the pa-

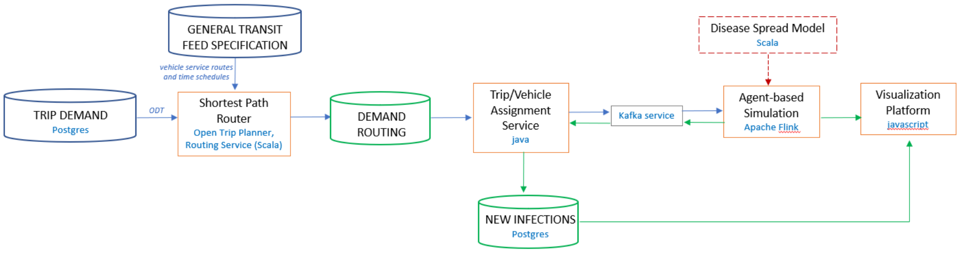

Figure 1: Transit assignment engine

tronage drop during the COVID-19 pandemic, we assume the demand to be 10% of the total, which aligns with the real-life observations [27, 29, 30] (see Table 1 in §1.3). The supply of services before and during COVID-19 was the same, so it was applied without changes.

Simply speaking, the transit assignment engine maps trip demand to transit service supply. In our case study, this means assigning a passenger to a train trip leg. Trip demand captured by smart cards, including tap-on and tap-off data with location GPS coordinates and timestamp, is used by the Shortest Path Router to calculate a set of time-dependent shortest paths for each travelling passenger using the classic Dijkstra algorithm. Each path represents one passenger trip, which may include multiple legs since passengers may transfer between services. Next, the paths are passed to the Trip/Vehicle Assignment Service module, where an attempt is made to assign each leg of the trip to a transit service vehicle (for example, a train) with some prior specified capacity and current occupancy. Transit service vehicles operate in accordance with a pre-defined schedule. The start time of the passenger trip needs to be matched with the scheduled transit vehicle operation. If a passenger cannot be assigned to a vehicle on a particular leg due to a vehicle capacity constraint, the next shortest path is requested from the Shortest Path Router and the assignment process is repeated until a feasible trip/vehicle assignment is found. Once all the feasible paths are defined and assigned to the vehicles, they are simulated in an agent-based environment. As the simulation is ongoing, the passengers are tracked and detailed dynamic outputs are collected for every agent (that is, a passenger), link (a link is the part of the trip between two consecutive stops/stations), stop/station, service vehicle, service line, and the whole network. Every time a passenger finishes their trip, a Disease Spread Model is triggered and this passenger is assigned a "status" corresponding with its outcome (that

is, during their trip, a passenger was infected or not). The information about new infections is sent to the Visualization Platform to be displayed on a dashboard. At the end of every simulated day and at the end of the simulation horizon, the total number of new infections is calculated and reported for final evaluation.

When it comes to the implementation of the transit assignment engine, traditional Java-based services backed by a relational database for transit assignment and trip event processing were developed, and an open source multi-modal trip planning library, Open Trip Planner (OTP), was used for the routing step in transit assignment. OTP was utilised as it includes implementations of both routing approaches (graph and adjacency list). It is robust and performs efficiently. In addition, a front-end was built using the modern JavaScript frameworks Vue.js, D3.js, and P5.js to visualise network activity at the vehicle level and provide aggregate origin-destination information in a dashboard format.

1.2 Transmission model on trains

At its core, the disease spread model on public transport network provides a probability of becoming infected for each susceptible passenger, based on the current and past travel of infectious passengers in the same spatial area. The model uses a number of simplifying assumptions, the most important being that we ignore any age-based effects (all agents are identical) and we assume homogeneity of mixing within the spatial area considered. We assume a half-carriage spatial area for our model, largely due to the upper and lower deck structure of the Sydney train carriages (Waratah design). Subsequently, our homogeneity of mixing assumptions means that passengers are equally divided amongst half-carriages of a train, and any spatial effects within a half-carriage are ignored.

The disease spread model for COVID-19 on trains has two transmission pathways, with quite different modelling approaches. The first pathway is direct (that is, person-to-person) transmission, for which we used the empirical attack rate on trains determined by Hu *et al.* [19] as a function of shared ride time (that is, exposure time). The second pathway is due to fomite (that is, person-to-surface-to-person) transmission, for which we use a more mechanistic approach, calibrated to recover the attack rate reported by Hu *et al.* [19]. These transmission pathways are combined into a single probability of infection for each susceptible passenger.

The overall probability of a susceptible passenger being infected from a single trip ($P(S \to E)$) is a combination of the probability of transmission

(p_i) from each infectious passenger (i), for each of the n infectious passengers the susceptible person has shared a half-carriage with, and from the probability of transmission from the half-carriage surfaces (p_c, which depends on i). The overall probability of a susceptible passenger becoming exposed as a result of their travel is the standard probability

$$P(S \to E) = 1 - (1 - p_c)\Pi_i^n(1 - p_i). \tag{1}$$

Both probabilities (p_i and p_c) are dependent on the mask wearing status of each agent of interest (that is, the susceptible being considered, and each of the infectious passengers).

The direct transmission probability, p_i, is based on the overall attack rate on a train found by Hu *et al.* [19]. It takes into account shared travel time (Δt_i for travel time with infectious passenger i) but not relative seat location, giving a probability of

$$p_{i0} = \frac{0.121 + 0.022\Delta t_i^2}{100}, \tag{2}$$

where the regression by Hu *et al.* [19] was for joint travel times of 8 hours or less. Note that Hu *et al.* [19] reported the attack rate as a percentage, which is why the probability has a different denominator here. We modify the probability p_i to take into account a number of factors, including: f_s for the proportion of the virus contributing to fomite transmission (p_s); m_i for the mask wearing status of each infectious passenger ($m_i = 1$ for those wearing a mask and 0 otherwise); and m_s for the mask wearing status of the susceptible passenger being considered (m_s). Subsequently, the direct transmission probability from infectious passenger p_i is given by

$$p_i(m_s, m_i) = (1 - m_s f_s)(1 - p_{s0}(m_i)) \min(p_{\max}, p_{i0}). \tag{3}$$

The $\min(p_{\max}, p_{i0})$ term is to ensure the probability stays within a sensible range in the event of the remote possibility of passengers sharing an especially long trip.

To calibrate the model, we relied on evidence reported in the literature. How mask wearing affects a susceptible passenger's probability of infection, through f_s, was calibrated to obtain the Odds Ratio of 0.22 reported by Wang *et al.*, 2020 [38]. Based on the work of Tang *et al.*2020 [34] and Chu *et al.*, 2020 [10], we use $p_{\max} = 0.16$.

Some ($p_s(m_i)$) of the virus shed by an infectious passenger who enters a half-carriage will be deposited on surfaces. How much depends on the mask wearing status of the infectious individual (m_i). We base our model of

the amount of deposited virus on the work by Atkinson and Wein, 2008 [1], and the estimates of decay on surfaces by Riddell *et al.* [31]. Due to these processes, the fomite transmission is modelled by considering the aggregated effect of infectious passengers on the half-carriage over time, and then we convert this into a probability of infection (p_c) via a standard dose response model. The overall probability of infection from surfaces (that is, the dose response) is given by

$$p_c(m_s) = p_{\max} \left[1 - \exp\left(-\frac{D_s(m_s)}{k}\right)\right], \tag{4}$$

where k is the response characteristic to an effective dose of COVID-19, which from Zhang and Wang, 2020 [44], is $k = 6.19 \times 10^4$; and the dose ($D_s(m_s)$) is the effect of the aggregated concentration of the virus on surfaces, taking into account the virus decay. The virus decay value comes from fitting an exponential decay model to the observed data reported by Riddell *et al.* [31].

The effective viral dose is given by

$$D_s(m_s) = \alpha(m_s) \sum_{\ell} \left[p_{s0} B \hat{G}_\ell \tau_\ell + \frac{1}{\mu_s}\left(C_{\ell 0} - p_{s0} B \hat{G}_\ell\right)(1 - \exp(-\mu_s \tau_\ell))\right], \tag{5}$$

where $C_{\ell 0}$ is the concentration on the carriage surfaces at the beginning of a link ℓ, B and $\alpha(m_s)$ are aggregations of constants, $\hat{G}_\ell$ is the effective number of un-masked infectious passengers on the half-carriage during a link ℓ, τ_ℓ is the duration of a link ℓ (note $\Delta t_i = \sum_\ell \tau_\ell$), and $\mu_s = \frac{0.04322}{24}$ is the surface decay rate of the virus from Riddell *et al.*, 2020 [31]. More specifically, $B = 2 \times \frac{10^5}{60\mu_s}$ is the aggregation of constants describing the surface concentration and dose from Zhang and Wang, 2020 [44]. The proportional constant for effective dose of the virus from a surface is given by the assumed functional form

$$\alpha(m_s) = (1 - m_s f_s)\alpha_0, \tag{6}$$

where $\alpha_0 = 0.28$ is the proportional constant for effective dose of virus from a surface in the absence of face masks, calibrated to obtain the attack rate from Hu *et al.*, 2020 [19] (Equation (2)).

Finally, the concentration of virus on the half-carriage surfaces at the end of a link j is given by

$$C_j = p_{s0} B \hat{G}_j \left(C_{j0} - p_{s0} B \hat{G}_\ell\right) \exp\left(-\mu_s \tau_j\right), \tag{7}$$

where p_{s0} is the proportion of virus shed by un-masked infectious passengers that ends up on surfaces. The value of p_{s0} is based on evidence that fomite transmission is a relatively unlikely route [14, 17, 26, 35], with Ferretti *et al.* [14] finding a speculative estimate of 10% of transmission being attributable to fomite transmission. We take this to be a rough estimate that $p_{s0} \approx 10\%$.

1.3 Seeding

Our primary disease spread model, as outlined in § 1.2, only considers transmission on the public transport network (and is specifically calibrated to trains). Furthermore, we do not track individuals between trips, so disease progression cannot be explicitly modelled. To overcome these limitations, and to keep the numbers of infectious passengers travelling on trains identical across simulations for comparability, we use a deterministic compartmental model to approximate the transmission dynamics in the general community.

We use the standard susceptible-exposed-infectious-recovered progression structure, with the exposed and infectious compartments repeated to better account for the distribution of time spent in those states (see, for example, [20]). We refer to this as an "SEEIIR" model for short.

As the transmission model is focused on, and calibrated to, transmission that is expected to occur on a train, it is necessary to consider community-wide transmission and how that will impact the transmission on a vehicle space unit (in our case, a space unit is a half-carriage). To this end, a simple population-level transmission model was used to seed the expected numbers of cases into the train network each day in the simulated time horizon (that is, 7 days). This model, "SEEIIR", follows a susceptible-exposed-infectious-recovered structure where: S is for susceptible, E for exposed (infected but not yet able to infect others), I for infectious (able to infect others), and R for recovered and immune. To better align with our epidemiological understanding of disease progression and to account for the distribution of time being spent "exposed" or "infectious" being more like an Erlang rather than an exponential distribution, we repeat those two compartments as shown in Figure 2.

The dynamics of transmission are then governed by the following system

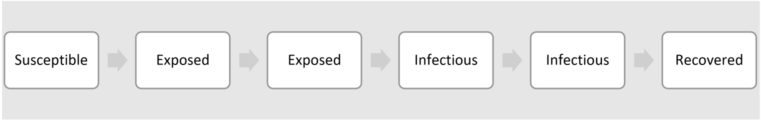

Figure 2: Community-wide "SEEIIR" transmission model, with the common susceptible-exposed-infectious-recovered structure. The repeated compartments better capture disease progression.

of ordinary differential equations (ODEs)

$$\frac{dS}{dt} = -\frac{\beta S(t)(I_1(t) + I_2(t))}{N}, \tag{8}$$

$$\frac{dE_1}{dt} = \frac{\beta S(t)(I_1(t) + I_2(t))}{N} - 2\sigma E_1(t), \tag{9}$$

$$\frac{dE_2}{dt} = 2\sigma E_1(t) - 2\sigma E_2(t), \tag{10}$$

$$\frac{dI_1}{dt} = 2\sigma E_2(t) - 2\gamma I_1(t), \tag{11}$$

$$\frac{dI_2}{dt} = 2\gamma I_1(t) - 2\gamma I_2(t), \tag{12}$$

$$\frac{dR}{dt} = 2\gamma I_2(t), \tag{13}$$

where the total population $N = S + E_1 + E_2 + I_1 + I_2 + R$ is constant, $\beta = 2.5\gamma$ is the transmission rate (that is, the value set to recover the expected $\mathcal{R}_0 = 2.5$) [6, 23], $1/\sigma = 3$ days is the average latent period (time from being infected to being able to infect others) [24, 46], and $1/\gamma = 13$ days is the average infectious period [24]. This model was implemented in Python 3.8, using the packages "matplotlib 3.3.2", "scipy 1.5.2", and "numpy 1.19.2", and numerical solutions used the "scipy.integrate.solve_ivp" integrator to solve the System of ODEs.

The model makes a number of simplifying assumptions, including: (1) everyone infected ends up with immunity; (2) a constant proportion of the infectious passengers travel as per before COVID-19; (3) super-infections are ignored; and (4) any interventions are ignored (at least explicitly). However, many of these limitations are strongly mitigated by the short time periods being considered in the full transport simulation (that is, the 7-day simulation horizon). We also do not explicitly consider asymptomatic infections, but this partially accounts for our assumption ignoring isolation and self-isolation. That is, people are less likely to self-isolate when asymptomatic.

As discussed in the paper, the starting point for the seeding process is 2000 infectious cases. This initial number is used in the SEEIIR model, which is based on the population of Sydney and a basic reproduction number of 2.5. We used estimates of the proportion of the population that commutes via public transport (approximately 20%, considering 2016, 2011, and 2006 Census records), and the proportion of commuters who use trains (approximately 50.9%), to arrive at an estimate of 10% of the population using trains in Sydney. This, combined with the estimate of 2000 initial infectious in the Sydney population of 5.73 million in 2019, resulted in the expected number of infectious individuals in the community, determined by running the Python implementation of Equations (8)–(13), and those travelling by train each day as reported in Table 1.

Table 1: Numbers of infectious in the community and estimated seeds for the simulation horizon (that is, 7 days) for the full train simulation modelling, rounded to the nearest integer.

Day	Total infectious in community	Infectious train passengers	Total train passengers
0	2000	200	1340708
1	1999	200	1386593
2	2044	204	1391588
3	2152	215	1409584
4	2312	231	1373788
5	2512	251	675665
6	2743	274	583467

Table 2 provides an overview of all the modelling parameters.

1.4 *Face mask wearing scenarios*

To determine the effectiveness of mask wearing, we had to answer two questions: (1) how mask wearing affects infectious passengers' shedding; and (2) how mask wearing affects susceptible passengers' probability of infection. The mask wearing status is then randomly conferred on passengers to recover the desired proportion of adherence to advice, with the effectiveness applied as appropriate and as follows.

To answer the first question, we use the filtration efficacy of a two-layer cloth mask described in Howard *et al.*, 2020 [18]. This was found to provide an 88-94% reduction in infection particle load and 80-90% filtration efficacy.

Table 2: Modelling parameters.

Symbol	Description	Value	Source
α_0	Proportional constant for effective dose of virus from a surface, in the absence of masks	0.0655	Calibrated to obtain attack rate from Hu *et al.*[19]
$\alpha(m_s)$	Proportional constant for effective dose of the virus from a surface	$(1 - m_s f_s)\alpha_0$	Assumed functional form
B	Aggregation of constants describing the surface concentration and dose	$2 \times \frac{10^5}{60\mu_s}$	[44]
f_i	How mask wearing affects infectious individuals' viral shedding	0.1	[18]
f_s	How mask wearing affects susceptible individuals' probability of infection	0.8	Calibrated to obtain the Odds Ration of 0.22 as reported by Wang *et al.*[38]
k	Response characteristic to an effective dose of COVID-19	6.19×10^4	[44]
p_{max}	Maximum probability of infection	0.16	[10, 34]
p_{s0}	Proportion of virus shed by infectious individuals that ends up on surfaces when no mask is worn	0.1	Value based on evidence fomite transmission is the relatively unlikely route [14, 17, 26, 35]
μ_s	Decay rate of virus on the surface(s)	$\frac{0.04322}{24}$ per hour	Exponential decay model calibrated to data from Riddell *et al.* [31]

In our modelling, we use 90% as it is within both of these bounds, and reduction in infection particle load is arguably the most important aspect.

Due to our one minus effect model structure, this gives us $f_i = 0.1$.

To answer the second question, we use the work by Wang *et al.*, 2020 [38] that identified an odds ratio of 0.22 for wearing a mask, compared to not wearing one. Its relationship to our effectiveness parameter (f_s) is not immediately apparent, and so we calibrated the value to recover the odds ratio from our model,

$$\text{OR} = \frac{\dfrac{\text{Number of infections wearing a mask}}{\text{not infected wearing a mask}}}{\dfrac{\text{infections not wearing a mask}}{\text{not infected not wearing a mask}}}. \tag{14}$$

We know that the expected number of cases will be

$$\mathbb{E} = P(S \to E)S_0. \tag{15}$$

Let the subscript m denote mask wearing and n no-masks, then the odds ratio equation becomes

$$OR = \frac{\dfrac{\mathbb{E}_m S_0}{S_0 - \mathbb{E}_m S_0}}{\dfrac{\mathbb{E}_n}{S_0 - \mathbb{E}_n S_0}} \tag{16}$$

$$= S_0 \frac{\dfrac{P(S \to E)_m}{1 - P(S \to E)_m}}{\dfrac{P(S \to E)_n}{1 - P(S \to E)_n}}. \tag{17}$$

The RHS is implicitly a function of f_s, and hence we can numerically find when the LHS=RHS. We used the scipy.optimize.fsolve and a Python 3.8 implementation of our model to determine how f_s varies with different p_{s0} values and different numbers of infectious passengers, I_0, shown in Figure 3. Given the tight range of $f_s \in [0.78, 0.84]$, we have chosen $f_s = 0.8$.

2 Sensitivity analysis

Sensitivity analyses are key to understanding how assumptions affect outcomes and recommendations. In the main paper, we covered the sensitivity analysis of how our results are affected by the proportion of virus shed that contributes to fomite transmission (p_{s0}) versus direct transmission ($1-p_{s0}$). Here, we explore how sensitive the calibration of parameter values is to

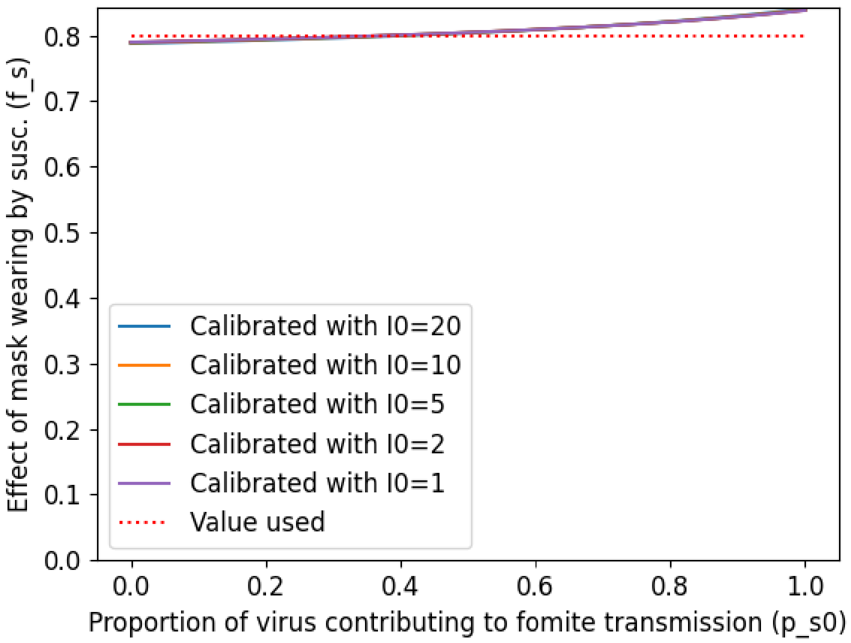

Figure 3: Sensitivity of the calibration of the effect of mask wearing by susceptible individuals, f_s, to the proportion of virus shed contributing to fomite transmission, p_{s0}, for different numbers of infectious passengers, I_0. Note that this calibration is insensitive to the number of infectious passengers on the half-carriage, I_0, with the lines completely overlapping.

variables of interest and determine the number of repeats required for consistent variance given the highly stochastic nature of the processes we are simulating.

2.1 Dose response calibration

As discussed in §1.2, we have assumed that the Hu *et al.* [19] attack rate is the ground truth, and have calibrated the fomite model accordingly. Here we show how sensitive those calibrations are to the proportion of virus shed contributing to fomite transmission (p_{s0}), the number of infectious passengers (I_0), and the duration of the mutual link (τ_ℓ).

Figure 4 depicts the sum of squared error between a simple simulation of 50 susceptible passengers on a half-carriage for times of Δt_i =

$[0.1, 0.2, 0.4, 0.6, 0.7, 0.95, 1.4]$ and the Hu *et al.* [19] attack rate, as a function of the dose response constant α_0. The travel times used reflect the distribution of the available data for the Sydney network. The minimum sum of squared error for α_0 for each of the different numbers of infectious passengers is provided in Table 3. Subsequently, we use $\alpha_0 = 0.28$ for all simulations.

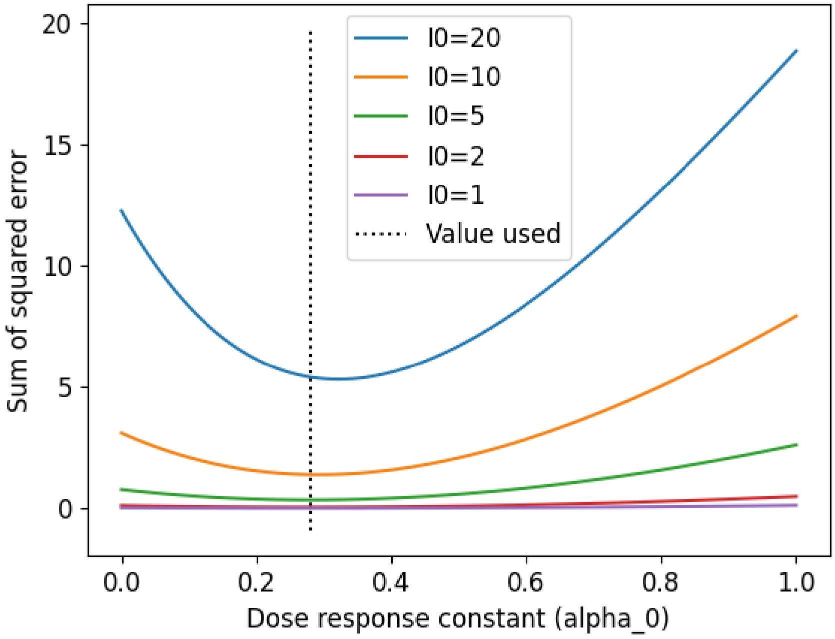

Figure 4: Sensitivity of the calibration of the dose response constant, α_0, to the proportion of virus shed contributing to fomite transmission, p_{s0}, for different numbers of infectious passengers, I_0. The dashed horizontal line is the value of α_0 used in all simulations presented in the paper.

Figure 5 depicts how the calibration of the dose response constant, α_0, is affected by the proportion of virus shed contributing to fomite transmission, p_{s0}. It shows that, with the exception of $p_{s0} = 0.0$ (that is, all virus shed contributes to direct transmission), the calibration is within a small range, with $\alpha_0 \in [0.27, 0.33]$. The $\alpha_0 = 0.28$ value used throughout is depicted by the horizontal dashed line, per the minimisation depicted in Figure 4. This aligns well with the smaller values of p_{s0} expected to reflect actual

Table 3: Optimal value of the dose response constant, α_0, to minimise the sum of squared error between number of infected and the Hu *et al.* [19] attack rate for different numbers of infectious passengers on the half-carriage with 50 susceptible passengers.

Total infectious passengers	Optimal α_0
20	0.32
10	0.29
5	0.28
2	0.27
1	0.27

COVID-19 transmission.

2.2 Number of repeats required to capture stochastic variance

Given the number of stochastic processes captured in our simulation, it is important to determine the minimum number of repeat simulations required to consistently capture the variance. Figure 6 depicts the average and 95% confidence intervals from different numbers of repeat simulations (that is, the whiskers represent 95% of the simulations). Here, the leftmost whisker plot incorporates the simulations from the runs represented in the rightmost whisker plot. So the "20" repeats includes all of the simulations from the "10", the "30" include all of the simulations from the "20", and so forth. We conclude from this that 60 repeats are sufficient to capture the stochastic variation. Therefore, the 100–200 repeats we have used throughout are sufficient to capture the stochastic variation.

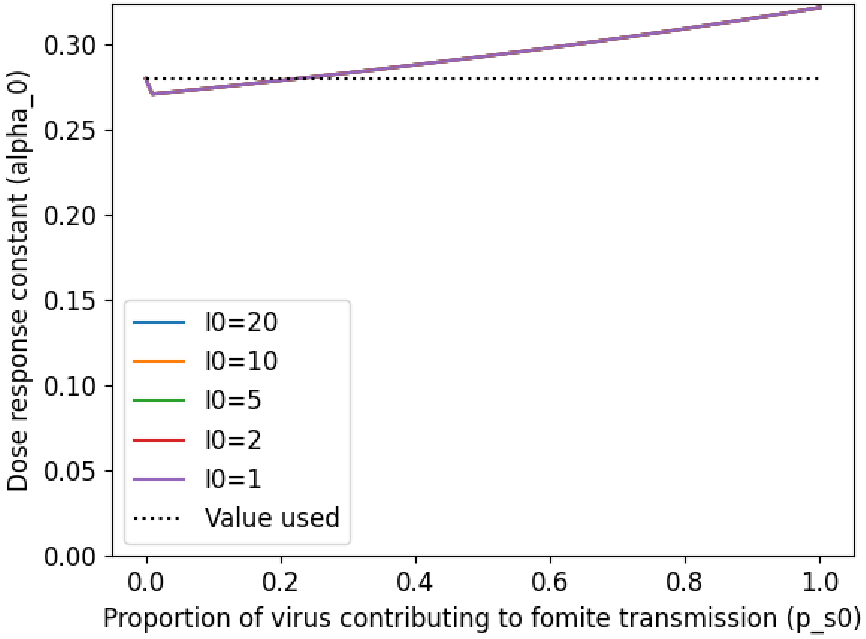

Figure 5: Sensitivity of the calibration of the dose response constant, α_0, to the proportion of virus shed contributing to fomite transmission, p_{s0}, for different numbers of infectious passengers, I_0. The dashed horizontal line is the value of α_0 used in all simulations presented in the paper.

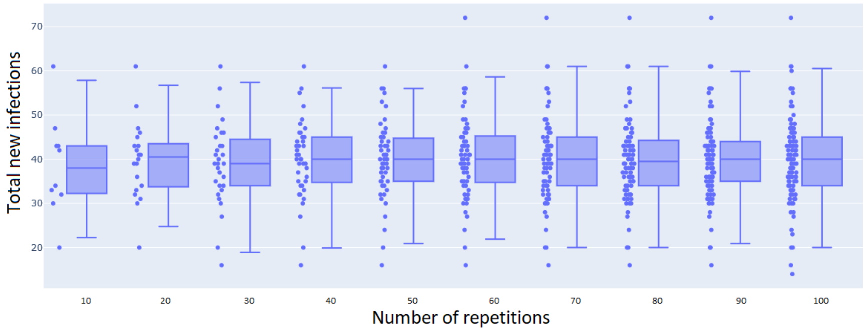

[a]

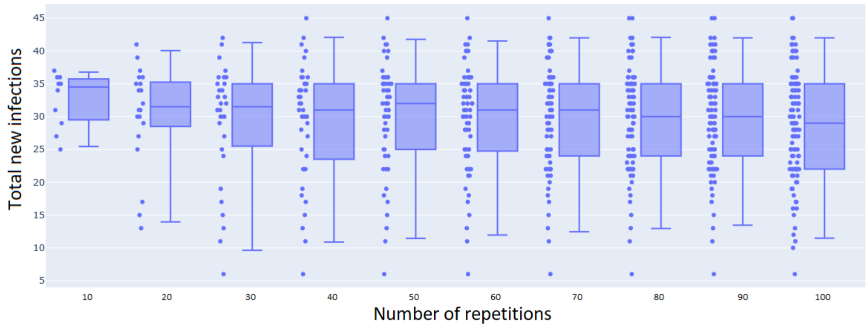
Total new infections
45
40
35
30
25
20
15
10
5
10
20
30
40
50
60
70
80
90
100
Number of repetitions

[b]

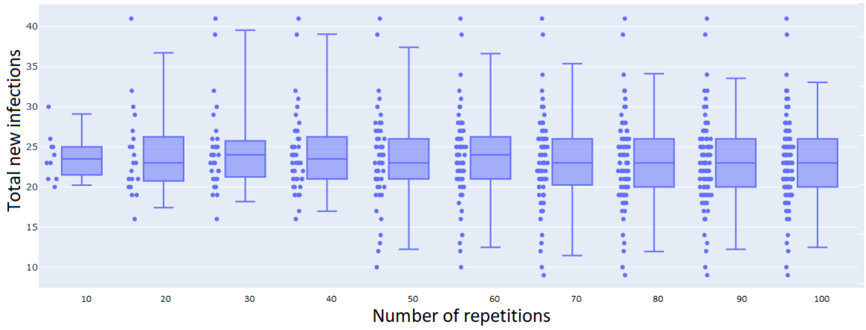
Total new infections
40
35
30
25
20
15
10
10
20
30
40
50
60
70
80
90
100
Number of repetitions

[c]

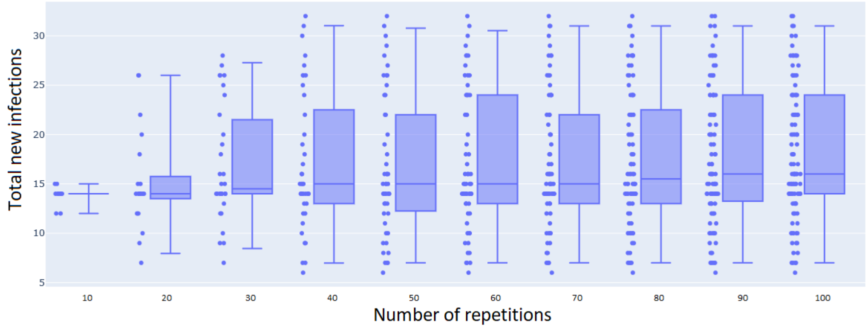
Total new infections
30
25
20
15
10
5
10
20
30
40
50
60
70
80
90
100
Number of repetitions

[d]

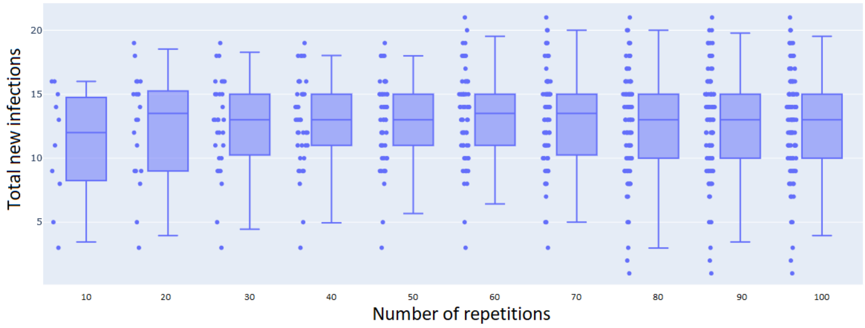

[e]

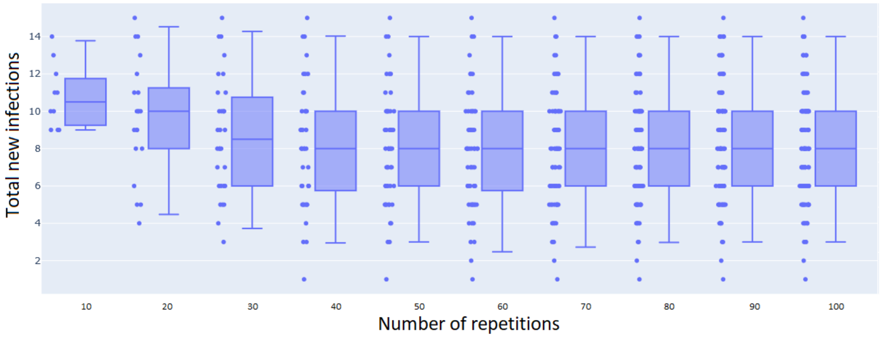

[f]

Figure 6: Effect of simulation number on stochastic variance for [a] baseline, [b] Mask_25, [c] Mask_50, [d] Mask_75, [e] Mask_80, and [f] Mask_100.

References

[1] Atkinson, M.P. and Wein, L.M., 2008. Quantifying the routes of transmission for pandemic influenza. *Bulletin of Mathematical Biology*, 70(3), pp.820-867.

[2] Australian Bureau of Statistics, 2008. *Public transport use for work and study.* https://www.ausstats.abs.gov.au/ausstats/subscriber.nsf/0/4028BBADB558AFFCCA25748E0012AEEF/$File/41020_2008_23.pdf (last accessed: 28 Jul 2021).

[3] Australian Bureau of Statistics, 2016. *Australian Social Trends.* https://www.abs.gov.au (last accessed: 28 Jul 2021).

[4] Australian Bureau of Statistics, 2016. *2016 Census QuickStats.* https://quickstats.censusdata.abs.gov.au/census_services/getproduct/census/2016/quickstat/1030 (last accessed: 28 Jul 2021).

[5] Australian Government. Department of Health, 2021. *Physical distancing for coronavirus (COVID-19).* https://www.health.gov.au/news/health-alerts/novel-coronavirus-2019-ncov-health-alert/how-to-protect-yourself-and-others-from-coronavirus-covid-19/physical-distancing-for-coronavirus-covid-19 (last accessed: 28 Jul 2021).

[6] Billah, M.A., Miah, M.M. and Khan, M.N., 2020. Reproductive number of coronavirus: A systematic review and meta-analysis based on global level evidence. *PloS One*, 15(11), p.e0242128. https://doi.org/10.1371/journal.pone.0242128

[7] Catching, A., Capponi, S., Yeh, M.T., Bianco, S. and Andino, R., 2021. Examining the interplay between face mask usage, asymptomatic transmission, and social distancing on the spread of COVID-19. *Scientific Reports*, 11(1), pp.1-11.

[8] CDC. "Community, Work, and School." Centers for Disease Control and Prevention, February 11, 2020. https://www.cdc.gov/coronavirus/2019-ncov/community/disinfecting-building-facility.html. (last accessed: 03 Aug 2021).

[9] Chowdhury, E.K., Khan, I.I. and Dhar, B.K., 2021. Catastrophic impact of Covid-19 on the global stock markets and economic activities. *Business*

and Society Review. https://doi.org/10.1111/basr.12219. (last accessed: 03 Aug 2021).

[10] Chu, D.K., Akl, E.A., Duda, S., Solo, K., Yaacoub, S., Schünemann, H.J., El-Harakeh, A., Bognanni, A., Lotfi, T., Loeb, M. and Hajizadeh, A., 2020. Physical distancing, face masks, and eye protection to prevent person-to-person transmission of SARS-CoV-2 and COVID-19: a systematic review and meta-analysis. *The Lancet*, 395(10242), pp.1973-1987.

[11] Coppola, P. and De Fabiis, F., 2021. Impacts of interpersonal distancing on-board trains during the COVID-19 emergency. *European Transport Research Review*, 13(1), pp.1-12. https://doi.org/10.1186/s12544-021-00474-6.

[12] Eikenberry, S.E., Mancuso, M., Iboi, E., Phan, T., Eikenberry, K., Kuang, Y., Kostelich, E. and Gumel, A.B., 2020. To mask or not to mask: Modeling the potential for face mask use by the general public to curtail the COVID-19 pandemic, *Infectious Disease Modelling*, 5, pp.293-308.

[13] Elbadawy, H.M., Khattab, A., Alalawi, A., Dakilallah Aljohani, F., Sundogji, H., Mahmoud, A.S., Abouzied, M., Eltahir, H.M., Alahmadey, Z., Bahashwan, S. and Suliman, B.A., 2021. The detection of SARS-CoV-2 in outpatient clinics and public facilities during the COVID-19 pandemic. *Journal of Medical Virology*, 93(5), pp.2955-2961. https://doi.org/10.1002/jmv.26819.

[14] Ferretti, L., Wymant, C., Kendall, M., Zhao, L., Nurtay, A., Abeler-Dörner, L., Parker, M., Bonsall, D. and Fraser, C., 2020. Quantifying SARS-CoV-2 transmission suggests epidemic control with digital contact tracing. *Science*, 368(6491), p.eabb6936. https://doi.org/10.1126/science.abb6936.

[15] Golin, A.P., Choi, D. and Ghahary, A., 2020. Hand sanitizers: A review of ingredients, mechanisms of action, modes of delivery, and efficacy against coronaviruses. *American Journal of Infection Control*, 48(9), pp.1062-1067. https://doi.org/10.1016/j.ajic.2020.06.182.

[16] Google. *Covid-19 Community Mobility Trends - Last updated 1 February 2021.* https://ourworldindata.org/covid-mobility-trends (last accessed: 28 Jul 2021).

[17] Greenhalgh, T., Jimenez, J.L., Prather, K.A., Tufekci, Z., Fisman, D. and Schooley, R., 2021. Ten scientific reasons in support of airborne transmission of SARS-CoV-2. *The Lancet*, 397(10285), pp.1603-1605. https://doi.org/10.1016/S0140-6736(21)00869-2.

[18] Howard, J., Huang, A., Li, Z., Tufekci, Z., Zdimal, V., van der Westhuizen, H.M., von Delft, A., Price, A., Fridman, L., Tang, L.H. and Tang, V., 2021. An evidence review of face masks against COVID-19. *Proceedings of the National Academy of Sciences*, 118(4), p.e2014564118. https://doi.org/10.1073/pnas.2014564118

[19] Hu, M., Lin, H., Wang, J., Xu, C., Tatem, A.J., Meng, B., Zhang, X., Liu, Y., Wang, P., Wu, G. and Xie, H., 2021. Risk of coronavirus disease 2019 transmission in train passengers: an epidemiological and modeling study. *Clinical Infectious Diseases*, 72(4), pp.604-610.

[20] Keeling, M.J., and Rohani, P., 2008. Modeling Infectious Diseases in Humans and Animals. Princeton University Press, 2008.

[21] Kamga, C. and Eickemeyer, P., 2021. Slowing the spread of COVID-19: Review of "Social distancing" interventions deployed by public transit in the United States and Canada. *Transport Policy*, 106, pp.25-36. https://doi.org/10.1016/j.tranpol.2021.03.014.

[22] Li, Y., Liang, M., Gao, L., Ahmed, M.A., Uy, J.P., Cheng, C., Zhou, Q., and Sun, C., 2021. Face masks to prevent transmission of COVID-19: A systematic review and meta-analysis, *American Journal of Infection Control*, 49(7), pp.900-906.

[23] Liu, Y., Gayle, A.A., Wilder-Smith, A. and Rocklöv, J., 2020. The reproductive number of COVID-19 is higher compared to SARS coronavirus. *Journal of Travel Medicine.* https://doi.org/10.1093/jtm/taaa021 (last accessed: 27 Jul 2021).

[24] Liu, Z., Magal, P., Seydi, O. and Webb, G., 2020. A COVID-19 epidemic model with latency period. *Infectious Disease Modelling*, 5, pp.323-337. doi: 10.1016/j.idm.2020.03.003

[25] Mackay, I.M, 2020. The Swiss cheese model of pandemic defense. *The New York Times.* `https://www.nytimes.com/2020/12/05/health/coronavirus-swiss-cheese-infection-mackay.html` (last accessed: 09 Aug 2021).

[26] Meyerowitz, E.A., Richterman, A., Gandhi, R.T. and Sax, P.E., 2021. Transmission of SARS-CoV-2: a review of viral, host, and environmental factors. *Annals of Internal Medicine*, 174(1), pp.69-79.

[27] Opendata Transport for New South Wales, 2021. *Opal Patronage.* https://opendata.transport.nsw.gov.au/dataset/opal-patronage (last accessed: 27 Jul 2021).

[28] O'Sullivan, M., 2017. Sydneysiders the nation's biggest users of public transport for work commute. *The Sydney Morning Herald.* https://www.smh.com.au/national/nsw/sydneysiders-the-nations-biggest-users-of-public-transport-for-work-commute-20171023-gz632p.html#: :text=Sydney%20tops%20the%20nation%20for%20the%20proportion%20of ,buses%2C%20trams%20or%20ferries%20for%20their%20daily%20commute (last accessed: 25 Jun 2021).

[29] Rabe, T., 2021. Sydney's public transport patronage at lowest levels since 1800s.*Sydney Morning Herald.* https://www.smh.com.au/national/nsw/sydney-s-public-transport-patronage-at-lowest-levels-since-1800s-20210719-p58b2v.html (last accessed: 19 Jul 2021).

[30] Rabe, T., and Singhal, P., 2020. The 51 million times Sydneysiders didn't tap on in March. *Sydney Morning Herald.* https://www.smh.com.au/national/nsw/the-51-million-times-sydneysiders-didn-t-tap-on-in-march-20200408-p54i4z.html (last accessed: 27 Jul 2021).

[31] Riddell, S., Goldie, S., Hill, A., Eagles, D. and Drew, T.W., 2020. The effect of temperature on persistence of SARS-CoV-2 on common surfaces. *Virology Journal*, 17(1), pp.1-7.

[32] Singh, P., Potlia, I., Malhotra, S., Dubey, H. and Chauhan, H., 2020. Hand sanitizer an alternative to hand washing — a review of literature. *Journal of Advanced Oral Research*, 11(2), pp.137-142. https://doi.org/10.1177/2320206820939403.

[33] Sun, C. and Zhai, Z., 2020. The efficacy of social distance and ventilation effectiveness in preventing COVID-19 transmission. *Sustainable Cities and Society*, 62, p.102390. https://doi.org/10.1016/j.scs.2020.102390.

[34] Tang, B., Bragazzi, N.L., Li, Q., Tang, S., Xiao, Y. and Wu, J., 2020. An updated estimation of the risk of transmission of the novel coronavirus (2019-nCov). *Infectious Disease Modelling*, 5, pp.248-255.

[35] Tang, J.W., Bahnfleth, W.P., Bluyssen, P.M., Buonanno, G., Jimenez, J.L., Kurnitski, J., Li, Y., Miller, S., Sekhar, C., Morawska, L. and Marr, L.C., 2021. Dismantling myths on the airborne transmission of severe acute respiratory syndrome coronavirus-2 (SARS-CoV-2). *Journal of Hospital Infection*, 110, pp.89-96. https://doi.org/10.1016/j.jhin.2020.12.022.

[36] Transport for New South Wales, 2021. *Train Patronage - Card Type*. https://www.transport.nsw.gov.au/data-and-research/passenger-travel/train-patronage/train-patronage-card-type (last accessed: 27 Jul 2021).

[37] Verschuur, J., Koks, E.E. and Hall, J.W., 2021. Observed impacts of the COVID-19 pandemic on global trade. *Nature Human Behaviour*, 5(3), pp.305-307. https://doi.org/10.1038/s41562-021-01060-5.

[38] Wang, Y., Tian, H., Zhang, L., Zhang, M., Guo, D., Wu, W., Zhang, X., Kan, G.L., Jia, L., Huo, D. and Liu, B., 2020. Reduction of secondary transmission of SARS-CoV-2 in households by face mask use, disinfection and social distancing: a cohort study in Beijing, China. *BMJ Global Health*, 5(5), p.e002794.

[39] Wikipedia, 2020. Transport for NSW patronage in Sydney by mode. https://en.wikipedia.org/wiki/Template:Transport_for_NSW_patronage_in_Sydney_by_mode (last accessed: 25 Jun 2021).

[40] Worby, C.J. and Chang, H.H., 2020. Face mask use in the general population and optimal resource allocation during the COVID-19 pandemic. *Nature Communications*, 11(1), pp.1-9.

[41] World Health Organization, 2020. Statement regarding cluster of pneumonia cases in Wuhan, China. Jan 9, 2020. https://www.who.int/china/news/detail/09-01-2020-who-statement-regardingcluster-of-pneumonia-cases-in-wuhan-china (last accessed: Feb 11, 2020).

[42] World Health Organization, 2020. Coronavirus disease 2019 (COVID-19) situation reports. https://www.who.int/

emergencies/diseases/novelcoronavirus-2019/situation-reports (last accessed: Feb 17, 2020).

[43] World Health Organization, 2020. WHO COVID-19 Dashboard. https://covid19.who.int/ (last accessed: 22 Jul 2021).

[44] Zhang, X., and Wang, J., 2020. Deducing the dose-response relation for Coronaviruses from COVID-19, SARS and MERS meta-analysis results. *MedRxiv*.

[45] Zhao, S., Lin, Q., Ran, J., Musa, S.S., Yang, G., Wang, W., Lou, Y., Gao, D., Yang, L., He, D. and Wang, M.H., 2020. Preliminary estimation of the basic reproduction number of novel coronavirus (2019-nCoV) in China, from 2019 to 2020: A data-driven analysis in the early phase of the outbreak. *International Journal of Infectious Diseases,* 92, pp.214-217.

[46] Yang, Z., Zeng, Z., Wang, K., Wong, S.S., Liang, W., Zanin, M., Liu, P., Cao, X., Gao, Z., Mai, Z. and Liang, J., 2020. Modified SEIR and AI prediction of the epidemics trend of COVID-19 in China under public health interventions. *Journal of Thoracic Disease*, 12(3), p.165. doi: 10.21037/jtd.2020.02.64.
